# The relationship between digital literacy and academic innovative behavior among STEM students: the mediating roles of knowledge-sharing intention and creative self-efficacy

**DOI:** 10.3389/fpsyg.2026.1937720

**Published:** 2026-09-24

**Authors:** Ruipeng Guo, Yongjun Feng

**Affiliations:** 1 Faculty of Education, Shaanxi Normal University, Xi'an, China; 2 College of Marxism, Xi'an University of Technology, Xi'an, China

**Keywords:** academic innovative behavior, creative self-efficacy, digital literacy, knowledge-sharing intention, STEM students

## Abstract

**Introduction:**

With the deep integration of digital technologies into higher education, digital literacy has become a key antecedent influencing college students' academic innovative behavior, yet its underlying mechanism remains insufficiently explored. Unlike previous studies that have focused primarily on a single mediating pathway, this study draws on knowledge creation theory and social cognitive theory to propose a parallel mediation model, aiming to simultaneously examine the unique roles of the “intention-based pathway,” represented by knowledge-sharing intention, and the “belief pathway,” represented by creative self-efficacy, in the effect of digital literacy on academic innovative behavior among STEM students, thereby revealing its structural transformation mechanism.

**Methods:**

Using stratified random sampling, this study selected 471 undergraduate students majoring in STEM disciplines from Xi'an University of Technology and employed structural equation modeling to analyze the data.

**Results:**

The results showed that (1) digital literacy was significantly and positively associated with knowledge-sharing intention and creative self-efficacy; (2) both knowledge-sharing intention and creative self-efficacy were significantly and positively associated with academic innovative behavior; and (3) both mediating pathways were significant and showed similar effect sizes, jointly accounting for 57% of the total effect, while the direct effect of digital literacy remained significant, indicating a partial mediating effect.

**Discussion:**

This study offers a more nuanced theoretical framework for understanding the process through which digital literacy contributes to academic innovative behavior and provides empirical evidence for a dual-pathway approach to digital literacy education and innovation-oriented talent cultivation in higher education.

## 1 Introduction

Since the beginning of the 21st century, digital technologies represented by artificial intelligence, big data, and cloud computing have been profoundly reshaping the landscape of higher education. Against this background, digital literacy, defined as individuals' ability to effectively use digital tools and resources to access, process, create, and communicate information, has been recognized by UNESCO as one of the core competencies for citizens in the 21st century (Law et al., 2018). In the context of higher education, digital literacy is not only related to whether students can adapt to digital learning environments, but also influences whether they can take an active role in knowledge production and academic innovation (Spante et al., 2018; Martin and Grudziecki, 2006).

As a key pillar of the national innovation system, STEM disciplines (science, technology, engineering, and mathematics) play an essential role in cultivating students' innovative abilities, which are closely associated with a country's scientific and technological competitiveness (Bybee, 2010). In the learning and research processes of STEM disciplines, digital tools are embedded in almost every stage: literature retrieval relies on academic databases (Holmes, 2016), data analysis depends on programming tools (Guzdial, 2015), and the presentation of research outcomes increasingly requires visualization technologies. Therefore, for STEM students, digital literacy is not merely a supporting skill, but a core productive capacity that is deeply embedded in the entire process of academic production (Falloon, 2020).

Although previous studies have confirmed the positive effects of digital literacy on learning performance and innovative behavior (Falloon, 2020; Shah et al., 2026; Siddiq et al., 2016; Hatlevik et al., 2015), a key theoretical question remains insufficiently addressed: through what psychological and behavioral mechanisms explain the association between digital literacy and academic innovative behavior? In recent years, some studies have attempted to introduce mediating variables, such as learning motivation (Shah et al., 2026), creative self-efficacy (Wu et al., 2025). However, these studies have generally focused on a single mechanism and have not fully revealed whether and how multiple pathways may operate in parallel. Although a single-mediation model can answer whether a mediating effect exists, it cannot explain whether mediating pathways of different natures coexist or which pathway plays a more prominent role. As a result, the structural process by which digital literacy is converted into academic innovation outcomes remains insufficiently understood.

To address these limitations, this study integrates knowledge creation theory and social cognitive theory to propose that digital literacy may influence academic innovative behavior through two distinct but potentially parallel pathways. The first is the intention-based pathway, represented by knowledge-sharing intention. In this pathway, digital literacy enhances students' willingness to share knowledge through digital platforms, while knowledge sharing facilitates the flow and recombination of knowledge, thereby fostering innovation (Wang and Noe, 2010). The second is the belief pathway, represented by creative self-efficacy. In this pathway, successful experiences associated with digital literacy strengthen students' confidence in their own innovative capabilities, thereby motivating innovative behavior. By simultaneously examining these two pathways within an integrated model, this study seeks not only to explain how digital literacy exerts its influence, but also to reveal the structural characteristics of its underlying mechanism, thereby providing more precise theoretical guidance for the design of digital literacy education pathways.

## 2 Literature review and hypothesis development

### 2.1 Digital literacy

The concept of digital literacy has continued to evolve with the iterative development of information technologies. Early studies mainly focused on the technical and operational dimensions, often equating digital literacy with computer operation skills or information retrieval abilities (Bawden, 2001). With the widespread adoption of Web 2.0 and mobile Internet, the meaning of digital literacy has been extended to multiple dimensions, including information evaluation, content creation, digital communication and collaboration, and cybersecurity (Van Laar et al., 2017). In the era of artificial intelligence, digital literacy has further expanded to include emerging elements such as an understanding of algorithmic logic and the ability to engage in human-machine collaboration (Long and Magerko, 2020).

In academic contexts, digital literacy is defined as an individual's comprehensive ability to effectively use digital technological tools and resources to acquire, screen, integrate, create, communicate, and disseminate academic information (Janssen et al., 2013). This definition includes not only an instrumental dimension, but also cognitive and social dimensions (Ng, 2012). Empirical research on college students' digital literacy has grown rapidly in recent years. For example, the review by Siddiq et al. (2016)) emphasized the multiple pathways through which digital literacy influences learning outcomes (Chen and Chiu, 2016).

### 2.2 Academic innovative behavior

The concept of “innovative behavior” originally emerged from the field of organizational behavior. (Scott and Bruce, 1994) defined employees' innovative behavior as the process through which individuals generate, promote, and implement useful new ideas in the workplace. Based on this conceptualization, (Janssen, 2000) further conceptualized innovative behavior as a three-stage process involving idea generation, idea promotion, and idea implementation. A systematic review of organizational creativity and innovation by Anderson et al. (2014)) further indicated that the multidimensional and context-dependent nature of innovative behavior has become widely recognized in the literature.

In higher education research, academic innovative behavior is defined as students' ability and behavior in generating novel and valuable academic ideas, research methods, or solutions during disciplinary learning and research training, and effectively putting these innovative ideas into practice (Lubart, 1996). Different from innovation in organizational contexts, students' academic innovative behavior has several distinctive characteristics. (1) As students are still in the process of learning and academic training, their innovation is more commonly reflected in the integration, reinterpretation, and rediscovery of existing knowledge (Beghetto and Kaufman, 2007). (2) It is strongly influenced by educational institutional factors, such as curriculum design and supervisor guidance (Hmelo-Silver et al., 2007). (3) It is usually manifested through course papers, research projects, graduation theses, and other academic outputs.

### 2.3 Knowledge-sharing intention

Knowledge-sharing intention refers to the degree to which individuals intend or plan to share their knowledge, experience, skills, and insights with others (Bock et al., 2005; Ryu et al., 2003). Knowledge sharing is a core concept in the field of knowledge management and is regarded as a key process in organizational knowledge creation and innovation (Alavi and Leidner, 2001). The formation of knowledge-sharing intention is influenced by multiple factors, including trust, norms of reciprocity, self-efficacy, and organizational climate (Lin, 2007; Hsu et al., 2007; Wasko and Faraj, 2005).

Knowledge creation theory (Nonaka et al., 2000) provides an important framework for understanding the relationship between knowledge sharing and innovation. This model proposes that knowledge creation proceeds through four stages: socialization, externalization, combination, and internalization. Within this cycle, knowledge sharing serves as the core mechanism of socialization and represents the starting point of the entire knowledge creation process. Without effective knowledge sharing, tacit knowledge cannot flow smoothly, and the generation of new knowledge becomes difficult.

In academic contexts, knowledge sharing is reflected in students' behavioral tendency to actively share learning materials, research experiences, technical methods, and other academic resources within academic communities (Cress and Kimmerle, 2008). With the development of digital technologies, the channels of knowledge sharing have expanded from face-to-face communication to various forms, such as online collaborative platforms and academic social networks (Majchrzak et al., 2013). Empirical studies have shown that knowledge sharing significantly promotes students' learning performance and innovative behavior (Koi-Akrofi et al., 2025).

### 2.4 Creative self-efficacy

Creative self-efficacy refers to individuals' belief in their ability to generate novel, original, and appropriate ideas or solutions according to task requirements. This concept originates from Bandura's social cognitive theory and represents a domain-specific extension of self-efficacy in the field of innovation. According to social cognitive theory, the main sources of self-efficacy include mastery experiences, vicarious experiences, verbal persuasion, and emotional and physiological states. Among these sources, mastery experiences are regarded as the most powerful source of efficacy information (Bandura, 1982).

In the field of innovation research, creative self-efficacy has been recognized as an important antecedent of innovative behavior (Carmeli and Schaubroeck, 2007). Individuals with higher creative self-efficacy are more likely to set challenging goals, demonstrate greater persistence when encountering difficulties, and translate innovative ideas into practice (Choi, 2004). The empirical study showed that employees' learning orientation indirectly affects employee creativity through the mediating role of creative self-efficacy.

### 2.5 Direct relationship between digital literacy and academic innovative behavior

The direct influence of digital literacy on academic innovative behavior can be understood from several perspectives. First, digital literacy enables students to efficiently access and integrate diverse sources of information, and this information-processing ability serves as a foundation for generating innovative ideas (Bawden and Robinson, 2009; Koltay, 2011). Second, digital tools provide technical support for students to translate innovative ideas into concrete academic outcomes (Claro et al., 2012). Finally, in STEM fields, frontier research often relies on complex digital tools and computational resources. Students who lack the corresponding digital literacy may therefore find it difficult to engage with these research frontiers (Voogt et al., 2013). Based on this, the present study proposes the following hypothesis:

H1: digital literacy is significantly and positively associated with STEM students' academic innovative behavior.

### 2.6 Mediating role of knowledge-sharing intention

Digital literacy may promote academic innovative behavior by enhancing knowledge-sharing intention, thereby forming a complete transformation chain of “ability–willingness–behavior.” Students with higher levels of digital literacy are more familiar with the operation and norms of digital collaborative tools, enabling them to share knowledge at lower cost and with greater efficiency. Meanwhile, students with higher digital literacy are more likely to experience the reciprocal value of information sharing when using digital platforms, thereby developing a stronger willingness to share knowledge (Lin, 2007; Hsu et al., 2007).

The mechanism through which knowledge sharing promotes innovation can be understood at two levels. At the individual level, by explaining and transmitting knowledge to others, knowledge sharers can deepen their own understanding and gain new perspectives and inspiration through interaction (Bock et al., 2005; Wasko and Faraj, 2005). At the group level, knowledge sharing facilitates the flow and recombination of knowledge, allowing knowledge and experience from different fields to intersect and integrate. Such knowledge recombination is an important source of innovation (Alavi and Leidner, 2001). Based on the above discussion, this study proposes the following hypothesis:

H2: knowledge-sharing intention mediates the relationship between digital literacy and academic innovative behavior among STEM students.

### 2.7 Mediating role of creative self-efficacy

Digital literacy may promote academic innovative behavior by enhancing creative self-efficacy, thereby forming a transformation pathway of “successful experience–competence belief–innovative behavior.” When students have higher levels of digital literacy, they are able to complete various digital academic tasks more efficiently and confidently (Hatlevik et al., 2015; Chen and Chiu, 2016). These successful task-completion experiences can strengthen students' general competence beliefs, which may further extend to innovation-related activities. In other words, the successful experiences brought about by digital literacy may be translated into students' confidence that they are capable of innovation (Tierney and Farmer, 2002; Gong et al., 2009).

The innovation process is often characterized by uncertainty and the risk of setbacks. When facing these challenges, individuals who believe in their ability to produce innovative outcomes are more likely to initiate the innovation process and persist when setbacks occur (Carmeli and Schaubroeck, 2007; Choi, 2004). Based on the above discussion, this study proposes the following hypothesis:

H3: creative self-efficacy mediates the relationship between digital literacy and academic innovative behavior among STEM students.

In summary, the framework developed in this study is illustrated in Figure 1.

**Figure 1:**
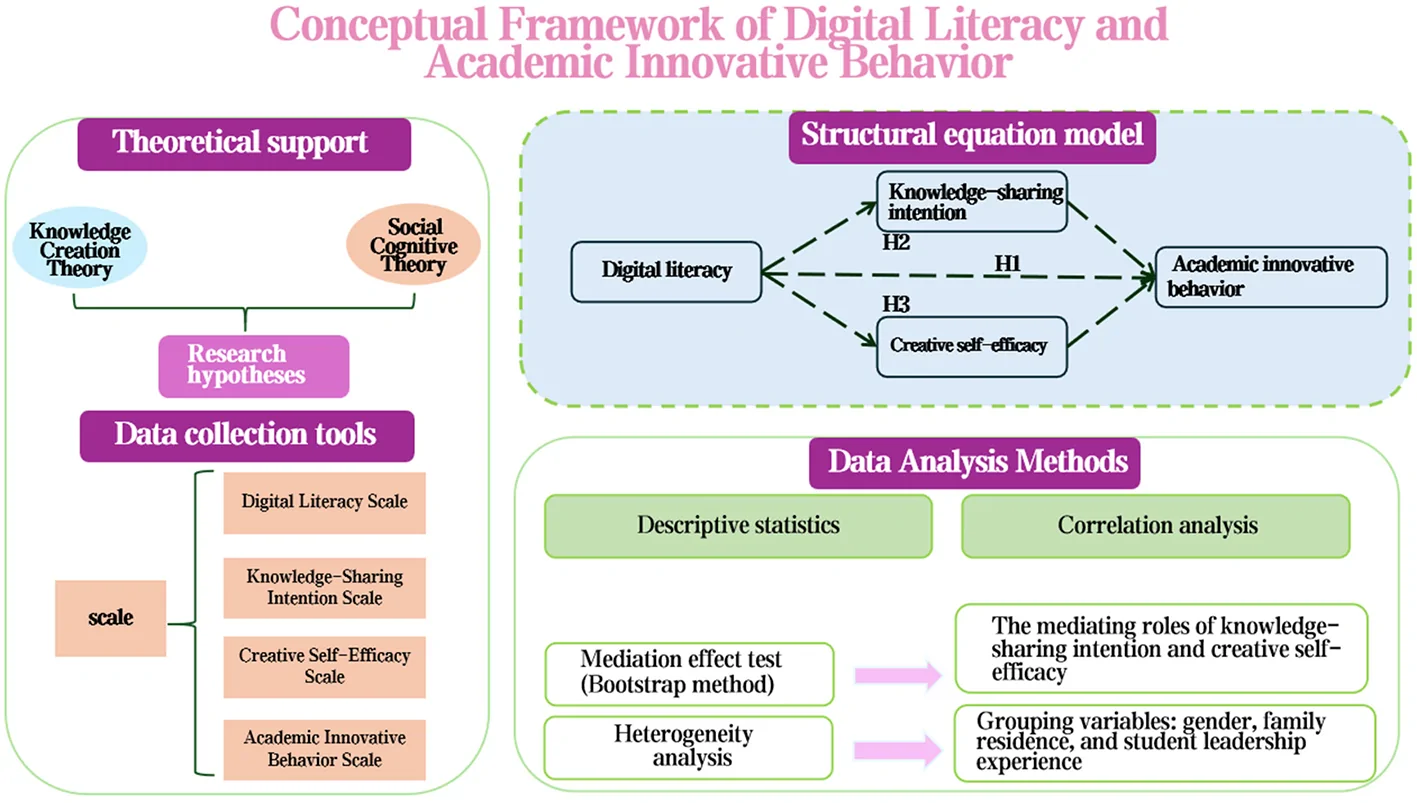
Parallel dual-mediation model.

## 3 Research methods

### 3.1 Participants and sampling

This study adopted a stratified random sampling method and conducted a questionnaire survey at Xi'an University of Technology from March to April 2025. Xi'an University of Technology is a comprehensive university with engineering as its dominant discipline. Its STEM disciplines, such as engineering and materials science, rank among the top 1% globally in ESI, and the university has a large population of STEM students as well as abundant digital teaching resources. The sampling procedure was as follows. First, all STEM majors at the university, including mechanical engineering, electronic engineering, computer science, civil engineering, materials science, and science-related disciplines, were divided into four strata according to academic year, from freshman to senior year. Second, within each stratum, classes were randomly selected in proportion to the number of students in each major. Finally, questionnaires were distributed to all students in the selected classes. A total of 500 questionnaires were distributed, and 471 valid questionnaires were collected, yielding an effective response rate of 94.2%.

This study was approved by the university ethics review committee. All participants provided informed consent after receiving a full explanation of the research purpose. To ensure data quality, this study used the Questionnaire Star online platform for questionnaire distribution and collection, and implemented several quality control measures.

First, screening items were included to accurately identify full-time undergraduate students majoring in STEM disciplines. Second, logical consistency checks and attention-check items were incorporated. Third, IP and device restrictions were enabled to prevent repeated submissions. Fourth, a minimum response time of no less than 200 seconds was set. Fifth, Data were collected using the Wenjuanxing platform, where all scale items were set as mandatory. No missing data were identified in the valid sample.

After data collection was completed, two researchers independently cleaned the data and excluded questionnaires with excessively short response times, obviously patterned responses, or responses that failed to meet the screening criteria. A total of 471 valid questionnaires were ultimately retained.

### 3.2 Measurement instruments

All scales used in this study were measured on a five-point Likert scale, ranging from 1 = strongly disagree to 5 = strongly agree. All scales were pre-tested with a pilot sample (*n* = 120) to examine their reliability and validity, and the wording of some items was revised based on the feedback obtained. In the present study, the Cronbach's α coefficients of all scales were above 0.855, indicating good reliability.

#### 3.2.1 Digital literacy scale

Participants' digital literacy was assessed using the Digital Literacy Scale developed by Wu (Ng, 2012) and adapted into Turkish by Üstündag et al. (2017)). The scale consists of 10 items and adopts a single-factor structure, explaining approximately 40% of the total variance. All items are rated on a five-point Likert scale, ranging from 1 = strongly disagree to 5 = strongly agree. There are no reverse-coded items in this scale. The Cronbach's α coefficient of the scale was 0.86 in the Turkish sample and 0.929 in the present study.

#### 3.2.2 Knowledge-sharing intention scale

Knowledge-sharing intention was measured using the scale developed by Bock et al. (2005)) Based on the theory of reasoned action, this scale measures individuals' willingness to share knowledge in organizational contexts. The scale has a single-factor structure and consists of five items, all rated on a five-point Likert scale. Representative items include “I am willing to share my professional knowledge and experience” and “I intend to share my knowledge in different groups and seminars.” The Cronbach's α coefficient of the scale was 0.93 in the original study and 0.877 in the present study.

#### 3.2.3 Creative self-efficacy scale

Creative self-efficacy was measured using the Creative Self-Efficacy Scale developed by Tierney and Farmer (2002)) and revised by Gong et al. (2009)) The original study by Tierney and Farmer used three items, and Gong et al. (2009)) added one additional item, forming the four-item version that is now widely used. The scale has a single-factor structure and is rated on a five-point Likert scale. Representative items include “I have confidence in my ability to propose new ideas at work” and “I am able to solve complex problems creatively.” The Cronbach's α coefficient of the scale ranged from 0.85 to 0.92 in the original studies and was 0.855 in the present study.

#### 3.2.4 Academic innovative behavior scale

Academic innovative behavior was measured using the Innovative Behavior Scale developed by Scott and Bruce (1994)) and revised by Janssen (2000)). Scott and Bruce conceptualized employees' innovative behavior as a unidimensional construct and initially developed a six-item scale. Based on this scale, Janssen further developed a nine-item version. Although the scale theoretically includes three dimensions, namely idea generation, idea promotion, and idea implementation, it has been used as a single-factor scale in many studies. The scale is rated on a five-point Likert scale. Representative items include “I search for and apply new working methods or technologies” and “I actively propose suggestions for improvement to optimize work processes.” The Cronbach's α coefficient of the original scale developed by Scott and Bruce was 0.89, and the Cronbach's α coefficient in the present study was 0.915. Previous higher education studies have successfully applied this scale to university student populations and demonstrated its reliability and validity in academic contexts. In the present study, the items were adapted to fit the academic context, for example, by replacing “work” with “study/research.” A pilot test (*n* = 120) was conducted to confirm the comprehensibility, reliability, and validity of the adapted scale in the present sample. The pilot results showed that Cronbach's α for all scales exceeded 0.85, indicating good reliability. Future research could further develop an innovative behavior scale specifically designed for academic contexts (Dai et al., 2022; Zhou, 2021).

#### 3.2.5 Control variables

To more accurately estimate the net effect of digital literacy on academic innovative behavior, this study controlled for the following demographic variables. These variables included gender (male = 1, female = 0), age as a continuous variable, family location (urban = 1, rural = 0), student leader experience (yes = 1, no = 0), and left-behind experience (yes = 1, no = 0). Left-behind experience refers to the experience of having one or both parents migrate for work for a cumulative period of more than 6 months before the participant reached the age of 18.

### 3.3 Data analysis

This study employed latent variable structural equation modeling (SEM) to test the hypothesized model. SPSS 27.0 was used for descriptive statistics, correlation analysis, and common method bias testing. AMOS 26.0 was used for the SEM analysis, with maximum likelihood estimation applied for parameter estimation. The model consisted of two components: a measurement model and a structural model. The measurement model used confirmatory factor analysis (CFA) to assess the relationships between the latent variables and their observed indicators, while the structural model examined the path relationships among the latent variables. Model fit was evaluated using the following criteria: χ^2^/df <3.0, CFI >0.90, TLI >0.90, RMSEA <0.08, and SRMR <0.08. Mediation effects were tested using a bias-corrected bootstrap procedure with 5,000 resamples, and 95% confidence intervals were calculated for the indirect effects. In the parallel mediation model, differences in the effect sizes of the two indirect pathways were inferred by comparing the overlap of their bootstrap confidence intervals and the decomposition of the total effect.

## 4 Research results

### 4.1 Reliability and validity analysis

The KMO values for all dimensions of the questionnaire were greater than 0.7, and Bartlett's test of sphericity was significant (*p* < 0.001), indicating sufficient correlations among the variables and supporting the suitability of the data for factor analysis.

Before testing the measurement model, the reliability and validity of the questionnaire were assessed. Reliability was evaluated using composite reliability (CR) and average variance extracted (AVE). Generally, CR values greater than 0.7 and AVE values greater than 0.5 indicate acceptable internal consistency among the measurement items. In this study, CR and AVE were calculated using AMOS 26.0. As shown in Table 1, all CR values exceeded 0.7 and all AVE values exceeded 0.5, indicating good internal consistency and acceptable reliability of the measurement items.

**Table 1:** Convergent validity results.

Path	Standardized factor loading	SE	*P*	CR and AVE
DL01←DL	0.783			(CR = 0.929, AVE = 0.568)
DL02←DL	0.736	0.055	0.000	
DL03←DL	0.782	0.054	0.000	
DL04←DL	0.716	0.054	0.000	
DL05←DL	0.697	0.055	0.000	
DL06←DL	0.796	0.051	0.000	
DL07←DL	0.784	0.056	0.000	
DL08←DL	0.750	0.055	0.000	
DL09←DL	0.723	0.053	0.000	
DL10←DL	0.761	0.053	0.000	
CSE01←CSE	0.766			(CR = 0.855, AVE = 0.596)
CSE02←CSE	0.730	0.066	0.000	
CSE03←CSE	0.791	0.067	0.000	
CSE04←CSE	0.800	0.065	0.000	
KS01←KS	0.766			(CR = 0.877, AVE = 0.589)
KS02←KS	0.737	0.063	0.000	
KS03←KS	0.808	0.061	0.000	
KS04←KS	0.774	0.060	0.000	
KS05←KS	0.750	0.062	0.000	
AI09←AI	0.676			(CR = 0.916, AVE = 0.548)
AI08←AI	0.705	0.074	0.000	
AI07←AI	0.765	0.071	0.000	
AI06←AI	0.744	0.074	0.000	
AI05←AI	0.752	0.071	0.000	
AI04←AI	0.727	0.072	0.000	
AI03←AI	0.804	0.073	0.000	
AI02←AI	0.758	0.072	0.000	
AI01←AI	0.722	0.073	0.000	

Validity was mainly assessed in terms of discriminant validity, which refers to the extent to which latent variables are empirically distinct from one another. The Fornell–Larcker criterion was used to evaluate discriminant validity by comparing the square root of the AVE for each latent variable with its correlations with other variables. The results showed that the square root of the AVE for each latent variable was greater than its correlations with the other variables (see Table 2), indicating good discriminant validity among the constructs.

**Table 2:** Discriminant validity results.

Variable	AI	KS	CSE	DL
AI	**0.741**			
KS	0.449^**^	**0.767**		
CSE	0.401^**^	0.151^**^	**0.772**	
DL	0.451^**^	0.375^**^	0.376^**^	**0.753**
AVE	0.548	0.589	0.596	0.568

^**^Correlation is significant at the 0.01 level (two-tailed). The bold values represent the square roots of the average variance extracted (AVE) for each construct.

### 4.2 Common method bias test

Harman's single-factor test was conducted by performing an unrotated exploratory factor analysis on all measurement items. The results showed that four factors had eigenvalues greater than 1, and the first factor explained 21.734% of the total variance, which was below the critical threshold of 40% (Podsakoff et al., 2003). This indicates that common method bias did not pose a serious threat in this study.

Second, a CFA common latent factor test was conducted. To further assess common method bias, a common method factor was added to the four-factor measurement model, allowing all observed indicators to load simultaneously on their corresponding construct factors and on the common method factor. Model fit was compared before and after adding the common method factor. The baseline four-factor model showed χ^2^/df = 1.114, CFI = 0.995, TLI = 0.994, and RMSEA = 0.016, whereas the model including the common method factor showed χ^2^/df = 1.023, CFI = 0.999, TLI = 0.999, and RMSEA = 0.007. The changes in CFI and TLI were ΔCFI = 0.004 and ΔTLI = 0.005, both below the recommended threshold of 0.10, while the change in RMSEA was only 0.009, below the recommended threshold of 0.05. These results indicate that adding the common method factor did not substantially improve model fit, suggesting that serious common method bias was not present in the data.

### 4.3 Descriptive statistics and correlation analysis

The valid sample consisted of 471 undergraduate STEM students from Xi'an University of Technology. The demographic characteristics of the sample are presented in Table 3.

**Table 3:** Table of sample characteristics.

Variable	Category	Frequency	Percentage
Gender	Female	175	37.2%
	Male	296	62.8%
Age	17	66	14%
	18	46	9.8%
	19	85	18%
	20	86	18.3%
	21	87	18.5%
	22	55	11.7%
	23	32	6.8%
	24	14	3%
Family residence	Rural	287	60.9%
	Urban	184	39.1%
Student leadership experience	No	296	62.8%
	Yes	175	37.2%
Left-behind experience	No	367	77.9%
	Yes	104	22.1%

Among the participants, 296 were male (62.8%) and 175 were female (37.2%). Their ages ranged from 17 to 24 years, with a mean age of 19.94 years (*SD* = 1.89). Regarding family residence, 184 students (39.1%) were from urban areas and 287 (60.9%) were from rural areas. A total of 175 students (37.2%) had student leadership experience, while 104 students (22.1%) had left-behind experience.

The means and standard deviations of all variables are presented in Table 4. The mean score for digital literacy was 3.35 (*SD* = 0.55), that for knowledge-sharing intention was 3.56 (*SD* = 0.62), that for creative self-efficacy was 3.47 (*SD* = 0.65), and that for academic innovative behavior was 4.36 (*SD* = 0.51), indicating that all four variables were at a moderately high level. The relatively high mean score for academic innovative behavior (4.36) may be related to the fact that the sample was drawn from an engineering-oriented university, where students have more opportunities to participate in research projects and disciplinary competitions. However, it may also be influenced by social desirability bias or the generalized nature of self-report measures, and therefore should be interpreted with caution.

**Table 4:** Means and standard deviations of variables.

Variable	*N*	Minimum	Maximum	Mean	Standard deviation
Gender	471.00	0.00	1.00	0.63	0.48
Age	471.00	17.00	24.00	19.94	1.89
Family location	471.00	0.00	1.00	0.39	0.49
Student leader experience	471.00	0.00	1.00	0.37	0.48
Left-behind experience	471.00	0.00	1.00	0.22	0.42
DL_mean	471.00	1.10	4.70	3.35	0.55
KS_mean	471.00	1.80	5.00	3.56	0.62
CSE_mean	471.00	1.75	5.00	3.47	0.65
AI_mean	471.00	2.78	5.00	4.36	0.51

As shown in Table 5, digital literacy was significantly and positively correlated with knowledge-sharing intention (*r* = 0.375, *p* < 0.01), creative self-efficacy (*r* = 0.376, *p* < 0.01), and academic innovative behavior (*r* = 0.451, *p* < 0.01). Knowledge-sharing intention was also significantly and positively correlated with academic innovative behavior (*r* = 0.449, *p* < 0.01), as was creative self-efficacy with academic innovative behavior (*r* = 0.401, *p* < 0.01). The correlation coefficients among the variables were all positive and reached a moderate level, providing preliminary support for the subsequent mediation effect test. Notably, although the correlation between knowledge-sharing intention and creative self-efficacy was significant (*r* = 0.150), its magnitude was relatively low, suggesting that the two constructs may have a certain degree of relative independence in content.

**Table 5:** Correlations among variables.

Variable	Gender	Age	Family Location	Student leader experience	Left-behind experience	DL_mean	KS_mean	CSE_mean	AI_mean
Gender	1								
Age	0.101^*^	1							
Family location	−0.06	0.01	1						
Student leader experience	−0.036	0.032	0.195^**^	1					
Left-behind experience	−0.025	−0.047	−0.174^**^	0.078	1				
DL_mean	−0.084	−0.091^*^	−0.089	0.123^**^	−0.146^**^	1			
KS_mean	0.006	−0.014	−0.038	0.024	−0.044	0.375^**^	1		
CSE_mean	−0.088	0.03	−0.019	−0.016	−0.096^*^	0.376^**^	0.150^**^	1	
AI_mean	−0.155^**^	0.008	−0.078	−0.022	0.004	0.451^**^	0.449^**^	0.401^**^	1

^*^Correlation is significant at the 0.05 level (two-tailed).

^**^Correlation is significant at the 0.01 level (two-tailed).

The multicollinearity test showed that the absolute values of the correlation coefficients among the explanatory variables were all below 0.7, and the VIF values ranged from 1.02 to 1.45 (Table 6), which were far below the critical threshold of 10. These results indicate that there was no serious multicollinearity problem among the variables.

**Table 6:** Multicollinearity test results.

Variable	Gender	Age	Family location	Student leader experience	Left-behind experience	DL_mean	KS_mean	CSE_mean
VIF	1.030	1.030	1.112	1.093	1.091	1.429	1.167	1.184
1/VIF	0.971	0.971	0.899	0.915	0.917	0.700	0.857	0.845

### 4.4 Structural equation modeling and mediation effect test

This study used structural equation modeling (SEM) to test the hypothesized parallel mediation model. The model fit indices reached acceptable standards: χ^2^/df = 1.114, GFI = 0.945, CFI = 0.995, and RMSEA = 0.016 (see Table 7), indicating that the theoretical model fit the observed data well.

**Table 7:** Results of model fit evaluation.

Test specification	CMIN/DF	GFI	IFI	CFI	RMSEA
Acceptance value	1–3	>0.90	>0.90	>0.90	<0.06
Results	1.114	0.945	0.994	0.995	0.016

The standardized path coefficients of the model were as follows: the path coefficient from digital literacy to knowledge-sharing intention was β = 0.416 (*p* < 0.001), and the path coefficient from digital literacy to creative self-efficacy was β = 0.423 (*p* < 0.001). After controlling for the mediating variables, the path coefficient from knowledge-sharing intention to academic innovative behavior was β = 0.363 (*p* < 0.001), the path coefficient from creative self-efficacy to academic innovative behavior was β = 0.304 (*p* < 0.001), and the direct path coefficient from digital literacy to academic innovative behavior was β = 0.207 (*p* < 0.001) (see Table 8). All direct paths reached statistical significance.

**Table 8:** Path analysis results of the structural equation model.

Path relationship test	Estimate	S.E.	C.*R*.	*P*
KS←DL	0.416	0.057	7.895	^***^
CSE←DL	0.423	0.057	7.955	^***^
AI←KS	0.363	0.043	7.101	^***^
AI←CSE	0.304	0.043	6.033	^***^
AI←DL	0.207	0.046	4.035	^***^

^***^Indicates statistical significance at *p* < 0.001.

As shown in Table 9, the indirect effect through knowledge-sharing intention was β = 0.136 (95% CI [0.095, 0.190]), while the indirect effect through creative self-efficacy was β = 0.116 (95% CI [0.078, 0.169]). The bootstrap confidence intervals for both pathways did not include zero, indicating that both mediation pathways were statistically significant. The total indirect effect was 0.253, accounting for 57% of the total effect (0.439). The two indirect pathways accounted for 31% and 26% of the total effect, respectively, indicating that their effect sizes were broadly comparable.

**Table 9:** Bootstrap test results of mediating effects.

Parameter	Estimate	Lower	Upper	*P*	Percentage of total effect
IE1:DL → KS → AI	0.136	0.095	0.19	0	31%
IE2:DL → CSE → AI	0.116	0.078	0.169	0	26%
IE	0.253	0.19	0.331	0	57%
DE	0.187	0.097	0.286	0	43%
TE	0.439	0.35	0.548	0	

Bootstrap resampling was repeated 5,000 times; confidence intervals were calculated using the bias-corrected percentile method.

To further examine whether the two indirect effects differed significantly, a bootstrap procedure with 5,000 resamples was used to directly estimate the confidence interval for the difference between the two indirect effects. The difference between the indirect effect through knowledge-sharing intention and that through creative self-efficacy was 0.020, with a bootstrap 95% CI of [−0.032, 0.072]. Because this interval included zero, the two indirect effects did not differ significantly (*p* > 0.05), which was consistent with the observation that their proportions of the total effect were broadly similar.

The direct effect was 0.187 (95% CI [0.097, 0.286]), accounting for 43% of the total effect, and its confidence interval did not include zero. This indicates that digital literacy retained a significant direct association with academic innovative behavior after accounting for the two mediators, suggesting partial mediation by knowledge-sharing intention and creative self-efficacy.

### 4.5 Multi-group structural equation modeling analysis

To examine whether the parallel mediation model linking digital literacy to academic innovative behavior was stable across different groups, multi-group structural equation modeling (multi-group SEM) was conducted using gender, family residence, and student leadership experience as grouping variables. A stepwise procedure was adopted to test four nested models: configural invariance, metric invariance, structural covariance invariance, and residual invariance. Model comparisons were based on the Δχ^2^ test and the ΔCFI criterion. Measurement invariance was considered supported when ΔCFI ≤ 0.01.

(1) Gender groups. The sample was divided into a male group (*n* = 296) and a female group (*n* = 175). The configural invariance model showed an acceptable fit (χ^2^/df = 1.124, CFI = 0.988, TLI = 0.987, RMSEA = 0.016) (see Table 10), indicating that the model had the same basic structural form across the two gender groups. After constraining the factor loadings to be equal, Δχ^2^(24) = 18.503, *p* = 0.778 > 0.05, and ΔCFI = 0.001 ≤ 0.01, indicating invariance of factor loadings across the two groups. After further constraining structural covariances and residuals, Δχ^2^(30) = 21.767, *p* = 0.862 > 0.05, ΔCFI = 0.001 ≤ 0.01, and Δχ^2^(61) = 53.195, *p* = 0.751 > 0.05, ΔCFI = 0.001 ≤ 0.01 (see Table 11), respectively. None of these differences was statistically significant. These results indicate strict measurement invariance across gender groups.

**Table 10:** Table of multi-group analysis model fit (gender).

Model	χ^2^	DF	*p*	CMIN/DF	GFI	CFI	TLI	RMSEA
Unconstrained	775.822	690	0.013	1.124	0.899	0.988	0.987	0.016
Measurement weights	794.325	714	0.019	1.112	0.897	0.989	0.988	0.015
Structural covariances	797.589	720	0.023	1.108	0.896	0.989	0.989	0.015
Measurement residuals	829.017	751	0.025	1.104	0.894	0.989	0.989	0.015

**Table 11:** Table of measurement invariance tests (gender).

Model	Δχ^2^	ΔDF	*p*	ΔGFI	ΔCFI	ΔTLI
Measurement weights	18.503	24	0.777948977	−0.002	0.001	0.001
Structural covariances	21.767	30	0.862384306	−0.003	0.001	0.002
Measurement residuals	53.195	61	0.751167584	−0.005	0.001	0.002

(2) Family residence groups. The sample was divided into an urban group (*n* = 184) and a rural group (*n* = 287). The configural invariance model showed a good fit (χ^2^/df = 1.034, CFI = 0.997, TLI = 0.996, RMSEA = 0.008) (see Table 12), indicating the same basic structural form across the urban and rural groups. After constraining the factor loadings to be equal, Δχ^2^(24) = 26.817, *p* = 0.313 > 0.05, and ΔCFI = −0.001 ≤ 0.01. After constraining structural covariances, Δχ^2^(30) = 31.771, *p* = 0.378 > 0.05, and ΔCFI = 0.000 ≤ 0.01. After constraining residuals, Δχ^2^(61) = 55.199, *p* = 0.685 > 0.05, and ΔCFI = 0.001 ≤ 0.01 (see Table 13). None of the differences was statistically significant, indicating strict measurement invariance across family residence groups.

**Table 12:** Table of multi-group analysis model fit (family residence).

Model	χ^2^	DF	*p*	CMIN/DF	GFI	CFI	TLI	RMSEA
Unconstrained	713.205	690	0.262	1.034	0.904	0.997	0.996	0.008
Measurement weights	740.022	714	0.243	1.036	0.901	0.996	0.996	0.009
Structural covariances	744.976	720	0.252	1.035	0.9	0.997	0.996	0.009
Measurement residuals	768.404	751	0.322	1.023	0.898	0.998	0.998	0.007

**Table 13:** Measurement invariance tests (family residence).

Model	Δχ^2^	ΔDF	*p*	ΔGFI	ΔCFI	ΔTLI
Measurement weights	26.817	24	0.313	−0.003	−0.001	0
Structural covariances	31.771	30	0.378	−0.004	0	0
Measurement residuals	55.199	61	0.685	−0.006	0.001	0.002

(3) Student leadership experience groups. The sample was divided into students with leadership experience (*n* = 175) and those without such experience (*n* = 296). The configural invariance model showed an acceptable fit (χ^2^/df = 1.122, CFI = 0.988, TLI = 0.987, RMSEA = 0.016) (see Table 14). After constraining the factor loadings to be equal, Δχ^2^(24) = 22.554, *p* = 0.546 > 0.05, and ΔCFI = 0.000 ≤ 0.01. After constraining structural covariances, Δχ^2^(30) = 32.456, *p* = 0.347 > 0.05, and ΔCFI = 0.000 ≤ 0.01. After constraining residuals, Δχ^2^(61) = 63.896, *p* = 0.375 > 0.05, and ΔCFI = 0.000 ≤ 0.01 (See Table 15). None of the differences was statistically significant, indicating strict measurement invariance across groups defined by student leadership experience.

**Table 14:** Table of multi-group analysis model fit (student leadership experience).

Model	χ^2^	DF	*p*	CMIN/DF	GFI	CFI	TLI	RMSEA
Unconstrained	774.494	690	0.014	1.122	0.896	0.988	0.987	0.016
Measurement weights	797.048	714	0.016	1.116	0.893	0.988	0.988	0.016
Structural covariances	806.95	720	0.013	1.121	0.892	0.988	0.987	0.016
Measurement residuals	838.39	751	0.014	1.116	0.889	0.988	0.988	0.016

**Table 15:** Measurement invariance tests (student leadership experience).

Model	Δχ^2^	ΔDF	*p*	ΔGFI	ΔCFI	ΔTLI
Measurement weights	22.554	24	0.546	−0.003	0	0.001
Structural covariances	32.456	30	0.346	−0.004	0	0
Measurement residuals	63.896	61	0.375	−0.007	0	0.001

Overall conclusion. The results of the three multi-group SEM analyses consistently indicate that the parallel mediation model demonstrates strict measurement invariance across gender, family residence, and student leadership experience. This suggests that the scales used in this study have the same measurement structure and meaning across different demographic groups. After establishing measurement invariance, the key structural paths were further compared across groups, and no significant between-group differences were identified (all ΔCFI ≤ 0.001). These findings suggest that the mediating mechanism through which digital literacy is associated with academic innovative behavior via knowledge-sharing intention and creative self-efficacy is stable across different demographic groups.

## 5 Discussion

Based on knowledge creation theory and social cognitive theory, this study constructed and tested a parallel dual-mediation model of the effect of digital literacy on academic innovative behavior among STEM students. The results generally supported the theoretical hypotheses and provided new theoretical insights at multiple levels.

Direct effect of digital literacy. Digital literacy had a significant positive direct effect on academic innovative behavior (β = 0.187, *p* < 0.001), thereby supporting H1. This finding is consistent with previous research (Shah et al., 2026) and further confirms this relationship among STEM students. Academic research in STEM fields is highly dependent on data-driven empirical methods. From literature retrieval and data analysis to the presentation of research outcomes, digital tools are indispensable at every stage (Falloon, 2020). Therefore, digital literacy constitutes a fundamental condition for academic innovation among STEM students. Meanwhile, the direct effect accounted for 43% of the total effect, indicating that a larger proportion of the influence of digital literacy, namely 57%, was indirectly realized through other mechanisms. This highlights the necessity of examining mediating pathways.

Mediating effect of knowledge-sharing intention. This study found that knowledge-sharing intention played a significant mediating role between digital literacy and academic innovative behavior, with an indirect effect of 0.136, accounting for 31% of the total effect. The Bootstrap confidence interval did not include zero, thereby supporting H2. This finding is consistent with the core proposition of knowledge creation theory, which emphasizes that knowledge sharing is the core mechanism of socialization and the starting point of knowledge creation. The path analysis showed that digital literacy significantly enhanced students' knowledge-sharing intention (β = 0.416, *p* < 0.001), while knowledge-sharing intention, in turn, significantly promoted academic innovative behavior (β = 0.363, *p* < 0.001). This finding indicates that, in the STEM learning context, the value of digital literacy is partly realized by facilitating knowledge exchange and collaboration within academic communities. Unlike studies that focus solely on the ability to acquire knowledge, this study reveals the unique role of knowledge “sharing,” rather than merely knowledge “acquisition,” in the transformation toward innovation.

Mediating effect of creative self-efficacy. Creative self-efficacy played a significant mediating role between digital literacy and academic innovative behavior, with an indirect effect of 0.116, accounting for 26% of the total effect. The Bootstrap confidence interval also did not include zero, thereby supporting H3. This finding is consistent with the prediction of social cognitive theory. Digital literacy provides students with repeated mastery experiences, such as successfully completing complex digital academic tasks. These successful experiences can be transformed, through a generalization mechanism, into stable confidence in their own innovative capabilities, thereby driving innovative behavior. The effect of the knowledge-sharing intention pathway accounted for 31% of the total effect, while that of the creative self-efficacy pathway accounted for 26%. The two effect sizes were broadly comparable, and their confidence intervals showed substantial overlap, suggesting no statistically significant difference between them. This result indicates that, in the process through which digital literacy is translated into academic innovative behavior, behavioral and belief mechanisms may be equally important and jointly constitute the parallel foundation of this transformation.

Structural characteristics and theoretical implications of the two pathways. The core finding of this study is that both the intention-based pathway and the belief pathway were significant and showed comparable effect sizes. This result goes beyond previous studies that focused on a single mechanism, such as the mediating role of motivation or perceived support, and provides a more complete structural picture. Specifically, digital literacy can promote innovation by facilitating students' knowledge-sharing behavior, which represents the intention-based pathway. It can also drive innovation by strengthening students' confidence in their innovative capabilities, which represents the belief pathway. Both pathways are indispensable. This parallel structure suggests that even when students have a high level of knowledge-sharing intention, they may still struggle to sustain innovation when facing uncertainty and setbacks if they lack innovative beliefs. Conversely, if students have confidence but lack actual knowledge exchange and collaboration, their creative ideas may have limited opportunities to collide, develop, and be refined. This dual-pathway explanatory framework provides a more integrative theoretical starting point for future research.

The heterogeneity analysis of this study has important theoretical implications. Using multi-group structural equation modeling (multi-group SEM), the study confirmed that the mediation model linking digital literacy to academic innovative behavior through knowledge-sharing intention and creative self-efficacy demonstrated strict measurement invariance across groups defined by gender, family residence, and student leadership experience (ΔCFI ≤ 0.001). This finding suggests that the mediating mechanism is not specific to any particular student group but is broadly applicable across different groups. Regardless of gender, urban–rural background, or student leadership experience, digital literacy was associated with greater academic innovative behavior through enhanced knowledge-sharing intention or creative self-efficacy. From a practical perspective, these findings suggest that digital literacy education and strategies promoting knowledge sharing can be implemented broadly across the student population without fundamentally different intervention pathways based on gender or urban–rural background. However, given the tendency toward a stronger direct association among students with leadership experience, differentiated advanced support for this group may still be considered in program implementation.

## 6 Conclusion

### 6.1 Main conclusions

Based on a survey of 471 STEM students from Xi'an University of Technology, this study reached the following conclusions.

First, Digital literacy is significantly and positively associated with STEM students' academic innovative behavior (β = 0.207, *p* = 0.000), providing empirical support for the important role of digital competence in STEM academic innovation.

Second, knowledge-sharing intention played a partial mediating role between digital literacy and academic innovative behavior. Digital literacy promoted innovation by enhancing students' willingness to share knowledge, and this pathway explained 31% of the total effect.

Third, creative self-efficacy played a partial mediating role between digital literacy and academic innovative behavior. Digital literacy promoted innovation by improving students' creative self-efficacy, and this pathway explained 26% of the total effect.

Fourth, the two mediating pathways jointly explained 57% of the total effect of digital literacy on academic innovative behavior. Moreover, their effect sizes were broadly comparable, suggesting that the intention-based pathway and the belief pathway have equally important parallel roles in the transformation of digital literacy into academic innovation.

### 6.2 Theoretical contributions

The theoretical contributions of this study are mainly reflected in three aspects. First, this study extends digital literacy research from a single perspective of “technical competence” to a dual-pathway perspective of “behavior-belief.” By simultaneously examining the mediating roles of knowledge-sharing intention and creative self-efficacy within an integrated model, this study reveals the structural mechanism through which digital literacy is transformed into innovation output. Specifically, the two pathways showed comparable effects and operated in parallel, providing a more refined theoretical framework for understanding how digital literacy is translated into innovation output. Second, this study integrates knowledge creation theory and social cognitive theory into the context of digital education, and verifies that the two theoretical pathways can operate in parallel with comparable effects, thereby providing empirical support for cross-theoretical integration. Third, the heterogeneity analysis using multi-group structural equation modeling (multi-group SEM) showed that this mediating mechanism was not specific to any particular student group, but demonstrated broad applicability and stability across groups. Regardless of gender, urban–rural background, or student leadership experience, digital literacy was associated with greater academic innovative behavior through enhanced knowledge-sharing intention.

### 6.3 Practical suggestions

Based on the above findings, the following suggestions are proposed.

First, digital literacy education should move beyond the level of tool operation and adopt a dual design that emphasizes both “behavioral activation” and “belief cultivation.” At present, digital literacy education in universities often focuses mainly on teaching students how to use software. It is therefore suggested that collaborative projects based on digital platforms be introduced to stimulate students' willingness to share knowledge. At the same time, hierarchical tasks and successful learning experiences should be designed to enhance students' creative self-efficacy through mastery experiences.

Second, universities should create an academic atmosphere that encourages knowledge sharing. Given the significant mediating role of knowledge-sharing intention in promoting innovation, teachers should incorporate collaborative performance into course evaluation systems, establish cross-grade and interdisciplinary academic communication platforms, and provide institutional recognition for academic mutual assistance. In this way, the knowledge-sharing function of digital literacy can be fully released at the organizational level.

Third, the potential leadership role of students with leadership experience in digital collaborative learning should be explored while interpreting the statistical results cautiously. Multi-group structural equation modeling showed that student leadership experience did not significantly moderate the mediation pathway (ΔCFI = 0.000, *p* = 0.546). Therefore, universities may consider incorporating digital collaboration skills into existing student leadership training programs as a supplementary approach to enhancing the effectiveness of digital literacy education. However, student leadership experience should not be regarded as a prerequisite for translating digital literacy into innovation.

Fourth, numerical gender differences should be interpreted cautiously to ensure the broad accessibility of digital literacy education. Multi-group structural equation modeling showed that gender did not significantly moderate the path coefficients in the mediation model (ΔCFI = 0.001, *p* = 0.778). Therefore, large-scale gender-differentiated curriculum design is not warranted based on these non-significant differences. Universities should instead ensure that digital literacy education is broadly accessible to both male and female students.

### 6.4 Research limitations and future directions

This study has several limitations.

First, the cross-sectional design of this study does not allow causal relationships among the variables to be established. Although the hypothesized model was developed based on theoretical reasoning, the findings only reflect statistical associations rather than strict causal relationships. The interpretation of full or partial mediation should therefore be theoretically grounded rather than treated as evidence of causality. Future studies could employ longitudinal designs or experimental interventions to further examine the causal relationships among these variables.

Second, the sample was drawn from a science and engineering university in western China. Although this university is representative in the STEM field, the generalizability of the findings to comprehensive universities, eastern regions, or different national contexts still requires further validation across universities, regions, and countries.

Third, the data were based on students' self-reports, and academic innovative behavior was measured using an innovative behavior scale originally developed in an organizational context. Although this scale showed high reliability and validity, its measurement content is more oriented toward innovative behavioral tendencies rather than academic innovative behavior in the strict sense. This may lead to two potential problems. First, there may be some content overlap with the creative self-efficacy scale, as both involve self-evaluations related to “innovation,” which may weaken the discriminant validity between constructs. Second, the relatively high mean score may partly reflect social desirability bias. Future research should use multi-source data for triangulation and develop an academic innovative behavior scale that is more specifically tailored to academic contexts.

Fourth, the heterogeneity analysis could be further extended. This study used multi-group structural equation modeling to examine measurement invariance across groups defined by gender, family residence, and student leadership experience, providing a basis for cross-group comparisons. However, the study did not formally test a moderated mediation model, that is, whether these variables strengthen or weaken the indirect pathway from digital literacy to academic innovative behavior through knowledge-sharing intention. Future research could therefore employ moderated mediation analysis to directly test whether the indirect effects differ significantly across groups. In addition, no clear difference was observed between students with and without left-behind experience. This may be because the participants were already in higher education, where early digital inequalities may have been partly reduced. Future studies could incorporate more refined indicators, such as the duration and severity of left-behind experience, and examine its moderating role across different educational stages to better understand its potential influence.

## Data Availability

The original contributions presented in the study are included in the article/Supplementary material, further inquiries can be directed to the corresponding author.
