# Supplementary material for: The relationship between digital literacy and academic innovative behavior among STEM students: the mediating roles of knowledge-sharing intention and creative self-efficacy
Source: Front Psychol. 2026 Sep 24;17:1937720. doi: 10.3389/fpsyg.2026.1937720 (PMC13646172; doi:10.3389/fpsyg.2026.1937720)
Supplement: Supplementary file 1 [file Supplementary_file_1.pdf]

Supporting Information

The Relationship Between Digital Literacy and Academic Innovative Behavior Among STEM Students: The Mediating Roles of Knowledge-Sharing Intention and Creative Self-Efficacy

Ruipeng Guo[a, b*], Yongjun Feng[a]

The studies involving human/animal participants were reviewed and approved by Student Affairs Department, Xi'an University of Technology.

Ruipeng Guo

[a]: Faculty of Education, Shaanxi Normal University, Xi’an, China.

[b]: College of Marxism, Xi’an University of Technology, Xi’an, China.

Email: 106617854@qq.com

Yongjun Feng

[a]: Faculty of Education, Shaanxi Normal University, Xi’an, China.

E-mail: yjfeng@snnu.edu.cn

*** Correspondence:**

Ruipeng Guo, College of Marxism, Xi’an University of Technology,

No.5, Jinhua South Road, Beilin District,

Xi'an 710048, China.

Email: 106617854@qq.com

Questionnaire

Dear student, there are no right or wrong answers on this anonymous questionnaire. Please select "√" in the appropriate option to indicate that you are responding in accordance with your current circumstances. Thank you for your participation!

Part I. Basic Information：

1. Gender： □Male □Female 2. Current Semester： ______ 3. Major： ______ 4.Region： ______

5.Political Affiliation： ______

Part II: Questionnaire Scales

Number	Code	Item	Strongly Disagree	Disagree	Neutral	Agree	Strongly Agree
1	A1	I believe that using AI chatbots can help me complete tasks more efficiently.	1	2	3	4	5
2	A2	I find the AI chatbot interface intuitive and user-friendly.	1	2	3	4	5
3	A3	I believe that the information provided by the chatbot is reliable and trustworthy.	1	2	3	4	5
4	A4	I intend to use AI chatbots more frequently over the next three months.	1	2	3	4	5
5	A5	I feel that this AI chatbot is capable of understanding my emotional needs during use.	1	2	3	4	5
6	A6	I feel comfortable using this AI chatbot.	1	2	3	4	5
7	T1	The teacher's explanations were understandable and clear from the beginning, and I think they will stay that way.	1	2	3	4	5
8	T2	The teacher’s explanations were logical and well-structured from the beginning, and I think they will stay that way.	1	2	3	4	5
9	T3	The teacher used appropriate language and speech pace to facilitate our understanding from the beginning, and I think they will stay that way.	1	2	3	4	5
10	T4	The teacher’s explanations were well adapted to the students’ level to facilitate understanding from the beginning, and I think they will stay that way.	1	2	3	4	5
11	T5	The teacher encouraged us to self-assess and become aware of our successes and challenges from	1	2	3	4	5

		the beginning, and I think they will stay that way.					
12	T6	The teacher encouraged us to engage in a certain degree of autonomous learning in this subject from the beginning, so that we could become independent individuals in the future, and I think they will stay that way.	1	2	3	4	5
13	T7	From the beginning (based on the course syllabus), the teacher encouraged us to decide for ourselves how to approach learning this course, and I think they will stay that way.	1	2	3	4	5
14	T8	The teacher provided me with confidence and alleviated my anxiety when I felt confused or frustrated, and I think they will stay that way.	1	2	3	4	5
15	F1	I lack confidence when speaking English in foreign language classes.	1	2	3	4	5
16	F2	I experience panic when speaking without preparation in foreign language classes.	1	2	3	4	5
17	F3	Even when thoroughly prepared, I experience anxiety in foreign language classes.	1	2	3	4	5
18	F4	I feel more nervous and uneasy in foreign language classes than in other courses.	1	2	3	4	5
19	F5	I feel my heart racing when I am about to respond to a question.	1	2	3	4	5
20	F6	In foreign language classes, I am so nervous that I forget what I have studied.	1	2	3	4	5
21	G1	I actively think about applying foreign language knowledge to practical situations when taking online courses.	1	2	3	4	5
22	G2	I make specific plans to accomplish foreign language learning tasks, such as daily vocabulary targets.	1	2	3	4	5

23	G3	I review complex foreign language materials repeatedly until I grasp the key points.	1	2	3	4	5
24	G4	I actively seek additional foreign language learning materials, such as films, news, or podcasts.	1	2	3	4	5
25	G5	I enjoy exploring a new language when learning a foreign language online.	1	2	3	4	5
26	G6	I remain motivated to learn a foreign language even without teacher supervision.	1	2	3	4	5
27	G7	I feel satisfied when making progress, such as improving my pronunciation.	1	2	3	4	5
28	G8	I am interested in learning foreign languages online.	1	2	3	4	5
29	G9	I actively participate in discussion forums by asking or answering questions in a foreign language.	1	2	3	4	5
30	G10	I actively take responsibility for my language learning in group tasks.	1	2	3	4	5
31	G11	I actively peer-review assignments and provide improvement suggestions to classmates through online platforms.	1	2	3	4	5
32	G12	I actively offer help to classmates when they encounter language difficulties, such as explaining grammar points.	1	2	3	4	5
